# Knee arthroplasty: are patients' expectations fulfilled? A prospective study of pain and function in 102 patients with 5-year follow-up

**DOI:** 10.1080/17453670902805007

**Published:** 2009-02-01

**Authors:** Anna K Nilsdotter, Sören Toksvig-Larsen, Ewa M Roos

**Affiliations:** ^1^Research and Development Department, Halmstad Central HospitalHalmstadSweden; ^2^Department of Orthopedics, Clinical Sciences, Lund UniversityLundSweden; ^3^Institute of Sport Sciences and Clinical Biomechanics, University of Southern DenmarkOdenseDenmark

## Abstract

**Background and purpose** With an aging population expecting an active life after retirement, patients’ expectations of improvement after surgery are also increasing. We analyzed the relationship between preoperative expectations and postoperative satisfaction and self-reported outcomes with regard to pain and physical function after knee arthroplasty.

**Patients and methods** 102 patients (39 men) with knee osteoarthritis and who were assigned for TKR (mean age 71 (51–86) years) were investigated with KOOS, SF-36, and additional questions concerning physical activity level, expectations, satisfaction, and relevance of the outcome to the patient. These investigations took place preoperatively and postoperatively after 6 months, 1 year, and 5 years of follow-up.

**Results** Response rate at 5 years was 86%. In general, the patients’ preoperative expectations were higher than their postoperative ability. For example, 41% expected to be able to perform activities such as golfing and dancing while only 14% were capable of these activities at 5 years. Having high or low preoperative expectations with regard to walking ability or leisure-time activities had no influence on the KOOS scores postoperatively. 93% of the patients were generally satisfied 5 years postoperatively, while 87% were satisfied with the relief of pain and 80% with their improvement in physical function at that time.

**Interpretation** With an expanding population of mentally alert elderly, we can expect that great demands will be put on joint replacements. This study shows that patients have high preoperative expectations concerning reduction of pain. To a considerable extent, these expectations are fulfilled after one year. Expectations concerning demanding physical activities are not fulfilled to the same degree; however, most patients reported general satisfaction with the outcome indicating that satisfaction is not equivalent to fulfilled expectations. Preoperative counseling should include realistic information on outcomes concerning physical function and pain relief.

## Introduction

Osteoarthritis (OA) severely affects overall health and health-related quality of life (Dieppe 1999, Jones et al. 2000). The most effective treatment for patients with severe OA of the knee is total knee replacement (TKR) (Bachmeier et al. 2001, Nilsdotter et al. 2001). The primary indication for TKR is pain, which can be relieved within 1 week after surgery (Aarons et al. 1996).

It is well known that the concerns and priorities of patients differ from those of surgeons and that surgeons are more satisfied than patients with the results of TKR and THR (total hip replacement) (Lieberman et al. 1996, Bullens et al. 2001). Knowing this makes it even more important to analyze the patients’ opinions about the outcome.

Questions have been raised about the importance of patients’ preoperative expectations concerning the postoperative outcome (Mahomed et al. 2002). With an aging population expecting an active life after retirement, patients’ expectations of improvement in physical function after surgery will become increasingly important. Positive expectations are associated with better health outcomes (Mondloch et al. 2001). There is also evidence of a strong correlation between satisfaction and expectations; patients with positive expectations preoperatively are more often satisfied with the outcome (Ronnberg et al. 2007).

It is not always clear, however, what constitutes a realistic expectation. To shed light on this, one must ask the patients about their expectations in relation to their way of living. When we know about their expectations and the results of surgery, it is possible to decide whether the expectations were realistic or not. Identifying patients’ expectations has a number of benefits, including improvement of patient satisfaction (Barron et al. 2007).

The main aim of this study was to analyze the relationship between preoperative expectations and self-reported improvement of physical function and pain 6 months, 12 months, and 5 years after TKR. The second aim was to investigate the relationship between preoperative patient characteristics and satisfaction after TKR.

## Methods

### Patients

123 consecutive patients who were on the waiting list for a primary TKR, because of osteoarthritis (OA), at the Department of Orthopedics at Lund University Hospital, Sweden, were sent questionnaires by mail. Patients were recruited from December 1999 through April 2001. 21 patients were excluded: 13 underwent other operative procedures and 8 were not operated during the study period. Thus, preoperative data were available for 102 patients (63 women) with OA of the knee (Table 1).

**Table 1.** Patient characteristics and outcome

Patient characteristics	Preop. n = 102/102	6 months n = 94/102	12 months n = 87/94	5 years n = 80/93
Age mean (SD)	71 (8.0)	71 (8.2)	72 (8.0)	76 (7.9)
[range]	[51–86]			[59–90]
No. of men (%)	39 (38)	38 (40)	37 (42)	33 (41)
No. of women (%)	63 (62)	56 (60)	50 (58)	47 (59)
BMI (SD)				28.4 (3.6)
[range]				[17–39]
KOOS pain	38 (18)	79 (20)	84 (17)	79 (21)
KOOS ADL	41 (16)	78 (17)	82 (16)	73 (21)
KOOS sport/recreation	17 (22)	48 (33)	47 (30)	52 (28)
SF-36 BP^a^	30 (18)	69 (25)	72 (23)	63 (25)
SF-36 PF^b^	27 (15)	58 (21)	61 (21)	51 (24)

^a^ bodily pain;

^b^ physical function.

The study was approved by the Ethics Committee of Lund University (LU 458-99).

### Questionnaires

All questionnaires were mailed to the patients and returned by mail in prepaid envelopes. The questionnaires consisted of one disease-specific questionnaire (the Knee injury and Osteoarthritis Outcome Score (KOOS)), one generic questionnaire (the SF-36), and questions on the relevance of improvement of the following KOOS subscales: expectations, satisfaction, and patient characteristics. The patients received questionnaires preoperatively and after 6 months, 12 months, and 5 years. Longitudinal outcome data from KOOS and SF-36 have been reported elsewhere (Nilsdotter et al. 2009).

*KOOS.* The KOOS method of scoring is an extension of the Western Ontario and McMaster Universities Osteoarthritis Index (WOMAC) (Bellamy et al. 1988). KOOS was developed for use with younger and/or physically active patients with knee injury and/or osteoarthritis (Roos et al. 1998), but it is also valid for elderly patients with TKRs (Roos and Toksvig-Larsen 2003). KOOS is a 42-item self-administered, self-explanatory questionnaire that covers 5 patient-relevant dimensions: pain, other disease-specific symptoms, ADL function, sports and recreation function, and knee-related quality of life. The WOMAC pain questions are included in the KOOS subscale “pain”, the WOMAC sepsness questions are included in the subscale “other disease-specific symptoms”, and the WOMAC subscale “function” is equivalent to the KOOS subscale “ADL”. In comparison to WOMAC, the KOOS could be advantageous when assessing groups with high expectations of physical activity and when assessing long-term outcome (Roos and Toksvig-Larsen 2003).

*SF-36*. The SF-36 is a widely used generic measure of outcome (Ware and Sherbourne 1992). It has 8 domains: PF (physical function), RP (role-physical), BP (bodily pain), GH (general health), VT (vitality), SF (social functioning), RE (role-emotional), and MH (mental health). Each subscale is scored from 0 to 100, with 0 indicating extreme problems and 100 indicating no problems. The SF-36 is self-explanatory and takes about 10 min to complete. The Acute Swedish version of the SF-36 was used (Sullivan et al. 1995).

### Comorbidities

Patients were asked if they were currently under treatment by a doctor, or if they had been treated during the previous year, for any of following 11 conditions: back problems, lung disease, high blood pressure, heart disease, impaired circulation in the lower extremities, neurological disease, diabetes, cancer, ulcer, kidney disease, and impaired vision or eye disease. The number of co-morbidities were summed and dichotomized, ≥ 2 or < 2.

At the 5-year follow-up, the patients were asked whether they suffered from arthritis-related pain in general, pain in their hips, or pain in their operated or unoperated knee.

### Level of physical activity

Level of physical activity was assessed with regard to walking ability and leisure activities (Roos et al. 1999). Walking ability was assessed on an ordinal scale from 1 to 6: (1) need for crutches or other device for moving more than a few steps; (2) walking indoors only; (3) walking indoors and less than 1 km outdoors; (4) walking more than 1 km outdoors; (5) walking unlimited distances on even ground; (6) walking on uneven terrain without any distance limit.

Leisure activities were assessed on a scale from 0 to 6: (0) no household work: TV and reading only; (1) minimal household work, card games, sewing; (2) light yard work, light household work, shopping; (3) heavy yard work, heavy household work; (4) golf, dancing, hiking, water aerobics; (5) recreational sports; (6) competitive sports.

### Relevance

Preoperatively, when deciding to have the operation, questions were asked about the relevance of pain relief, reduction in other symptoms, improvement in activities of daily living (ADL) function, improvement in sports and recreational function, and improvement in knee-related quality of life (QOL) (i.e. improvement in the 5 different KOOS domains). Responses were graded on a 5-point Likert scale ranging from extremely important to not important at all.

### Expectations

Preoperatively, expectations were assessed in relation to walking ability and leisure activities and in relation to the 5 above-mentioned KOOS domains: pain, other symptoms, ADL function, sports and recreation functions, and knee-related QOL. Patients were also asked to estimate the time to postoperative recovery.

Expectations of walking ability were graded on a 6-point Likert scale from need for crutches or other devices to walking unlimited on uneven terrain. Expectations of leisure activity were graded on a 7-point Likert scale from “doing no household work” to “engaging in competitive sports”.

Expectations, in relation to the KOOS subscales, were graded on a 5-point Likert scale ranging from “much less” to “much more” for pain and other symptoms; and ranging from “much better” to “much worse” for ADL, sports and recreational activities, and knee-related quality of life.

Postoperatively, questions were asked regarding the change experienced in each of the 5 KOOS subscales. The answer options related to the subscales pain and other symptoms ranged from “much less” to “much more”, and for ADL, sports and recreational activities, and knee-related quality of life they ranged from “much better” to “much worse”.

### Satisfaction

Postoperatively, 1 question was asked about satisfaction with the result in general. The responses were graded on a 5-point Likert scale from “totally satisfied” to “very dissatisfied”.

5 other questions were asked about satisfaction in relation to the 5 domains of the KOOS (pain relief, symptom relief, improvement in ADL function, improvement in sport and recreational function, and improvement in knee-related quality of life). Responses were graded in the same way on a 5-point Likert scale from “totally satisfied” to “very dissatisfied”.

### Statistics

To describe the results, continuous outcomes are given as mean (SD) and range. Ordinal outcomes are given as percentages. To study the differences between preoperative expectations and fulfilled expectations, McNemar test was used. To study the possible influence of preoperative expectations on postoperative outcome, the patients were dichotomized in 2 groups: those having high expectations of improvement in walking ability and leisure activities and those having low expectations. For these comparisons, we used the Mann-Whitney U-test. Spearman’s rank correlation was used for correlations of preoperative expectations of pain relief and improvement in ADL function to age, BMI, and number of co-morbidities. The Mann-Whitney test was used for gender associations.

To investigate the possible influence of preoperative characteristics on postoperative satisfaction correlations were made between the preoperative characteristics: KOOS subscales pain and ADL, the SF-36 subscales PF, BP, and GH, age, BMI and number of co-morbidities and postoperative satisfaction in relation to the 5 KOOS domains using Spearman’s rank correlation test.

To study the difference in physical function (KOOS ADL and SF-36 PF) at the 5-year follow-up between those who reported low back pain, arthritis-related pain, pain in their hips, or pain in their knees, we used the Mann-Whitney U-test. Statistical analysis was done using SPSS version 14.1.

## Results

### Patients

At the 5-year follow-up, 9 patients had died and responses were available from 80/93 patients (86%) with a mean age of 76 years (Table 1). Preoperatively, the patients estimated their average time of recovery to be 4 (1–12) months, indicating that most patients expected full recovery already at the first follow-up 6 months after surgery.

### Expectations of walking ability and leisure activities

**Figure 1.**
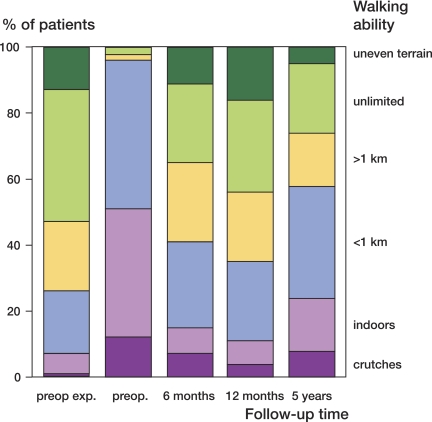
Breakdown of patients’ (n = 80) expectations preoperatively, the situation preoperatively, and outcome concerning walking ability. Crutches: the need for crutches or some other device to move more than a few steps; indoors: able to walk indoors; < 1 km: able to walk indoors and less than 1 km outdoors; > 1 km: able to walk more than 1 km; unlimited: unlimited walking on even ground; uneven terrain: unlimited walking on uneven terrain.

Expectations of walking ability were better fulfilled than expectations of leisure time activities. Preoperatively, 39% of the patients expected to have unlimited walking ability on even ground. The patients reported the best walking ability 12 months postoperatively, where 28% could walk without limitation on even ground. At 5 years, 21% could walk on even ground without any distance limitations (Figure 1).

**Figure 2.**
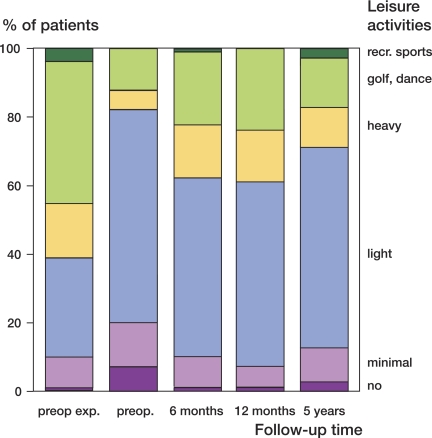
Breakdown of patients’ (n = 80) expectations preoperatively, the situation preoperatively, and outcome concerning leisure activities. No: no household work, only TV and reading; minimal: minimal household work, card games, and sewing; light: light yard work, light household work, shopping; heavy: heavy yard work, heavy household work; golf, dance: golf, dancing, hiking, water aerobics; recr sports: recreational sports.

In general, patients’ expectations about leisure activities were higher than their results at 1 and 5 years after operation. For example, 41% of the patients expected to be able to go dancing and golfing while only 24% reported being able to do so at the 1 year follow-up (Figure 2).

### Expectations of improvement in ADL and sport and recreational function

**Figure 3.**
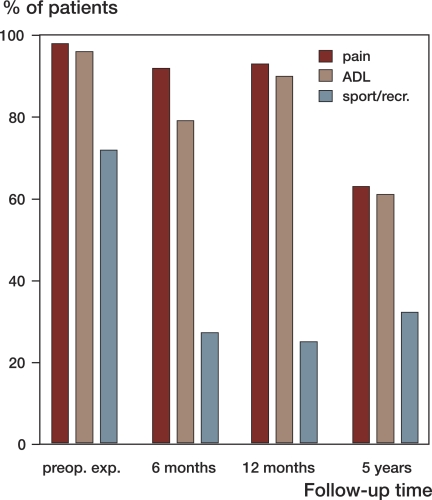
Percentages of patients (n = 80) with high expectations (much less or less pain; better or much better ADL, better or much better sport/recr) preoperatively and the percentages of patients reporting fulfilled expectations at 3 different follow-up times. ADL: activities of daily living; sport/recr: sport and recreational function; exp: expectations.

Questions concerning ADL function and sport and recreational function were extremely important or very important for 94% and 51% of the patients, respectively. Expectations were higher for ADL function than for sport and recreational function: 96% expected much better or better ADL function while 72% expected much better or better sport and recreational function. There was a greater gap between expectations and reported improvement for sport and recreational function than for ADL at the 12-month follow-up. The best improvement in ADL function was reported at 12 months when 90% reported much better or better ADL function (p = 0.3) while only 25% experienced improvement in sport and recreational function (p < 0.001). At 5 years, 61% experienced improved ADL function (p < 0.001) and 32% experienced improved sport and recreational function (p < 0.001) (Figure 3).

### Expectations of pain relief

Questions concerning pain were extremely or very important for 94% of the patients.

Preoperatively, 98% of the patients expected much less or less pain postoperatively. Similarly to physical function, walking ability, and leisure-time activities, the best outcome regarding self-reported pain was seen at the 12-month follow-up when 93% experienced much less or less pain (p = 0.3). After 5 years, 63% experienced much less or less pain than preoperatively (p < 0.001) (Figure 3).

### Level of expectation and outcome in physical function

Having higher expectations of ability regarding leisure activities was associated with a higher postoperative level of leisure activity and better walking ability. Those who had higher expectations of their ability in leisure activities reported improvement in leisure activities (from level 2 to level 3) (p < 0.001) and improvement in walking ability (from level 3 to level 4) (p = 0.01) at the 5-year follow-up. No single patient achieved a higher level of physical activity than previously anticipated. There were, however, no clinically or statistically significant differences in perceived functional difficulties as measured by KOOS scores for ADL function or for sport and recreational function at 5 years postoperatively when comparing those with high and low expectations of improvement in leisure activities, or when comparing those with higher or lower expectations of walking ability.

### Influence of age, BMI, gender, and co-morbidities on expectations

There was a weak correlation between the preoperatively expected change in pain and age (r_s_ = 0.18) and the preoperatively expected change in ADL function and age (r_s_ = 0.20), where older patients had lower expectations. The correlations between the expected change in pain and BMI or number of co-morbidities (r_s_ = 0.10, r_s_ = 0.22) and between the expected change in ADL function and BMI or number of co-morbidities (r_s_ = 0.06, r_s_ = 0.003), were generally even weaker.

### Postoperative satisfaction

**Figure 4.**
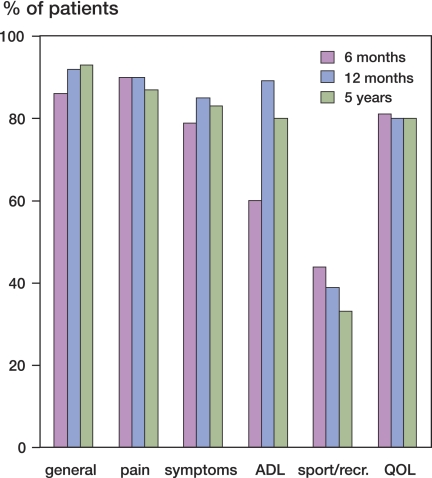
Percentage of patients who reported satisfaction (extremely satisfied, very satisfied) at the follow-ups 6 months, 12 months, and 5 years after TKR. The first (left-hand) block shows general satisfaction and the others show specific satisfaction in relation to the 5 KOOS subscales.

Most patients (93%) were generally satisfied with the result of the operation 5 years postoperatively. The lowest records of specific satisfaction with pain relief, symptom relief, functional improvement, or quality of life were reported for sport and recreational function where only one-third of the patients were totally or quite satisfied at five years (Figure 4).

Less preoperative pain was associated with higher postoperative satisfaction for all KOOS subscales. Likewise, better preoperative function and better general health were associated with higher postoperative satisfaction in the KOOS domains pain, symptoms, and ADL. There was a correlation between lower BMI and higher satisfaction with sport and recreation function. No other associations were found between age, BMI, and comorbidities to satisfaction with improvement in the KOOS domains (Table 2).

**Table 2.** Correlation between preoperative variables and postoperative satisfaction

Satisfaction	KOOS pain	KOOS ADL	SF-36 PF	SF-36 BP	SF-36 GH	Age	BMI	Co-morbidity
Pain relief	0.26^a^	0.24^a^	0.06	0.18	0.27^a^	0.18	–0.13	–0.13
Relief of symptoms	0.35^a^	0.25^a^	0.03	0.23^a^	0.29^a^	0.15	–0.14	–0.12
Improvement in ADL	0.37^a^	0.35^a^	0.21	0.29^a^	0.37^a^	0.16	–0.11	–0.15
Sport/recreational improvement	0.24^a^	0.22	0.10	0.16	0.23	0.20	–0.30	–0.10
Knee-related QL improvement	0.29^a^	0.14	0.11	0.16	–0.31^a^	0.19	–0.03	–0.13

ADL – activity of daily living, PF – physical function, BP – bodily pain, GH – general health

Spearman’s rank correlation test. Results are given as r_s_.^a^ p < 0.05.

### Musculoskeletal comorbidity

At the 5-year follow-up, as many as one-third of the patients reported musculoskeletal pain (Table 3). There were significant differences in KOOS ADL scores and SF-36 PF scores at the 5-year follow-up between those who reported low back pain, arthritis-related pain in general, and pain in their knees (data not shown).

**Table 3.** Percentage of patients at the 5-year follow-up who suffered from musculoskeletal pain. 6 patients were operated bilaterally (data not shown)

Musculoskeletal co-morbidity	
Low back pain	35%
Arthritis-related pain	28%
Hip pain (left hip)	16%
Hip pain (right hip)	20%
Knee pain, operated knee	9%
Knee pain, unoperated knee	21%
Knee pain bilaterally	14%
No knee pain	56%

## Discussion

We found that patients’ high expectations concerning pain relief after TKR were to a great extend fulfilled while their expectations about demanding physical activities were not fulfilled to the same degree.

### Expectations

It is known that, despite their age, many knee patients participate in a wide range of recreational activities such as dancing and golfing and are disappointed with the results of joint replacement (Weiss et al. 2002). These findings are clearly supported by our data where almost all expected improvement in ADL function and as many as three-quarters expected improvement in sport and recreational function.

The expectations of patients concerning walking ability and ADL function are fulfilled to a greater extent postoperatively than their expectations concerning more demanding physical activities such as sport and recreation. We found a clear increase in patients’ experience of unfulfilled expectations of physical function between 1 and 5 years, even though there was improvement compared to preoperative function. Our results probably reflect the new generation of patients with OA who expect to continue with an active life for a long time after the operation. Patients may also be influenced by the marketing of new prostheses, the implication being that the patient will achieve a high level of physical activity provided he/she just gets the right implant.

The expectations of improvement in physical function were almost as high as the expectations of pain relief (96% and 98%, respectively). These data are striking, as the primary indication for joint replacement is still pain. The expectations concerning pain were very high, and they were almost fulfilled 1 year after TKR in our study. Thus, expectations concerning pain appear to be realistic—which contrasts with the unrealistic expectations concerning demanding physical activities.

Previous studies have also shown a great difference between these two outcome variables, where the degree of pain relief expected has been considerably greater. In the study by Mahomed et al. (2002), 40% of patients expected no limitations to their usual activities and 76% expected no pain after surgery. That study and our study may not be entirely comparable because questions about expectations were asked in different ways. On the other hand, our results suggest that pain relief alone is not enough to satisfy TKR patients; they also require better physical function.

The strength of our study is that we assessed the outcomes in a manner that included demanding activities and expectations concerning such activities by using KOOS as outcome measurement. It is also clear that these questions are relevant for the patients. It is notable that even in this elderly population, questions concerning sport and recreational function were relevant for more than half of the patients.

Our data make it clear that joint replacement is excellent in terms of treatment of pain but it does not fulfill the patients’ expectations of improved sport and recreational function. Assuming that it is desirable to achieve such an ambition, we must make better efforts concerning postoperative rehabilitation—such as the way in which we treat patients after total hip replacement when we know that postoperative rehabilitation improves the outcome (Unlu et al. 2007).

One can assume that the expectations concerning physical function and pain would be related to age. Our study, as well as the study by Mahomed et al. (2002), did not find any significant statistical correlation between expectations concerning physical function, pain, and age, gender, BMI, or number of co-morbidities. Consequently, we can count on men as well as women in older age having high expectations of the procedures we offer in the future.

Obviously, it is impossible to have any opinion about the patients’ expectations preoperatively without asking for it. There appears to be no relationship between overall severity of preoperative disability (and also background factors) and the patient’s preoperative expectations (Haddad et al. 2001, Eisler et al. 2002, Mahomed et al. 2002).

### Satisfaction

The general satisfaction with the procedure was remarkably high 5 years after TKR. However, when the question is broken down further and different domains of satisfaction are separated, another picture presents itself. The patients were least satisfied with their sport and recreational function, which could be a problem in the future with an active elderly population. There was also a tendency of declining satisfaction after 1 year. The decline was more obvious for physical function than for pain. This is an additional reason to assess the outcome with KOOS, where demanding physical activities and leisure activities are taken into consideration.

In our study, it appears that general satisfaction mainly concerned pain relief and improved ADL functions, even though patients had high expectations of sport and recreational function. The satisfaction with pain relief was obvious early in the postoperative process while satisfaction with improvement in ADL function was most pronounced after one year.

When analyzing the correlation between postoperative satisfaction and preoperatively reported status, we found a significant importance of the self-reported preoperative pain and general health. Individuals with a moderately painful knee before operation and satisfactory general health appear to have been be the most satisfied patients.

### Expectations and satisfaction

Fulfilled expectations are not necessary equivalent to satisfaction. The patients in our study were satisfied with the outcome despite unfulfilled expectations. Satisfaction is a complex concept, and it is influenced by many factors but especially by expectations and outcome (Mancuso et al. 1997). General satisfaction is, to our knowledge, even more complex and should not be used as the primary outcome. It is too blunt an instrument for this purpose.

### Follow-up time

Our study is one of the few studies dealing with patient expectations and satisfaction to have a follow-up period of as long as 5 years. In a previous study, it was stated that to determine the true success of a procedure it is critical to evaluate it over time and that there is a lack of data regarding patients’ expectations for this type of procedure over time (Lieberman et al. 2003). Our study revealed a decline in outcome after 1 year. The feelings that expectations have been fulfilled and feelings of satisfaction with the procedure also decline. This is noteworthy, because the final clinical assessment often takes place 1 year after TKR.

In summary, we found that patients’ expectations concerning pain, ADL function, and walking ability were realistic but their expectations concerning sport and recreational function and leisure activities were not. It is important that orthopedic surgeons and physiotherapists inform their patients preoperatively in a way that will give them more realistic expectations of the results of procedures such as total knee replacement.
